# Characterization of starvation-induced autophagy in cerebellar Purkinje cells of pHluorin-mKate2-human LC3B transgenic mice

**DOI:** 10.1038/s41598-020-66370-6

**Published:** 2020-06-15

**Authors:** Juan Alejandro Oliva Trejo, Isei Tanida, Chigure Suzuki, Soichiro Kakuta, Norihiro Tada, Yasuo Uchiyama

**Affiliations:** 1 Department of Cellular and Molecular Neuropathology, Juntendo University Graduate School of Medicine, Bunkyo-Ku Tokyo, Japan; 2 Research Institute for Diseases of Old Age, Juntendo University School of Medicine, Bunkyo-Ku Tokyo, Japan

**Keywords:** Macroautophagy, Cellular neuroscience

## Abstract

We generated a new transgenic mouse model that expresses a pHluorin-mKate2 fluorescent protein fused with human LC3B (PK-LC3 mice) for monitoring autophagy activity in neurons of the central nervous system. Histological analysis revealed fluorescent puncta in neurons of the cerebral cortex, hippocampus, cerebellar Purkinje cells, and anterior spinal regions. Using CLEM analysis, we confirmed that PK-LC3-positive puncta in the perikarya of Purkinje cells correspond to autophagic structures. To validate the usability of PK-LC3 mice, we quantified PK-LC3 puncta in Purkinje cells of mice kept in normal feeding conditions and of mice starved for 24 hours. Our results showed a significant increase in autophagosome number and in individual puncta areal size following starvation. To confirm these results, we used morphometry at the electron microscopic level to analyze the volume densities of autophagosomes and lysosomes/autolysosomes in Purkinje cells of PK-LC3 mice. The results revealed that the volume densities of autophagic structures increase significantly after starvation. Together, our data show that PK-LC3 mice are suitable for monitoring autophagy flux in Purkinje cells of the cerebellum, and potentially other areas in the central nervous system.

## Introduction

In neurons of the central nervous system (CNS), macroautophagy (henceforward, autophagy) has long been established as an essential process for preserving homeostasis and preventing cell death. Under normal conditions, basal autophagy prevents defective protein aggregation and accumulation, and participates in organelle turnover^1–3^. In general, autophagy is considered a beneficial system, but in some cases neuronal autophagy may prove to be detrimental. For example, it has been reported that inducing autophagy in a brain ischemia and hypoxia model is associated with a worse prognosis^4^. Therefore, basic questions about how autophagy is regulated need to be addressed before targeting autophagy as a medical therapy.

The relationship between autophagy dysregulation and neuronal health has been under intense investigation. Mouse models of autophagy deficiency that target the essential autophagy genes atg5^5^, atg7^6^ and atg9a^7^ in neurons, exhibit severe pathological phenotypes. These mice display severe growth retardation, ataxia and a reduced lifespan. Intracellular changes in neurons feature ubiquitylated protein accumulation and cell death^5–7^. These models have shown that the neuronal axon is particularly vulnerable to autophagy deficiency, exhibiting dysregulated development^7^ and pathological changes^5^. This is important because axonal degeneration is recognized as a key turning point that leads to neurodegeneration^8–10^. In humans, dysregulated autophagy has been associated with important neurodegenerative diseases, such as Alzheimer’s^11,12^, Parkinson’s^3,12,13^, Huntington’s^11,13^, Niemann-Pick diseases^14^ and Amyotrophic Lateral Sclerosis^3,15^.

At present, there are many methods and mouse models for monitoring autophagy both *in vitro* and *in vivo*. Mizushima *et al*. used a Green Fluorescent Protein (GFP) probe attached to an LC3B protein to establish the first mouse model for monitoring autophagy activity *in vivo*^16^. They showed that autophagy signals fluctuate in peripheral tissue cells following a period of nutrient starvation, although GFP-LC3 puncta were not detected in the brain^16^. However, recent studies have shown that autophagy is induced in Purkinje cells and in other neurons of GFP-LC3 mice^17,18^. In recent years, at least 6 new mouse models have been developed for monitoring autophagy^19,20^. Among these models, two have been developed specifically for monitoring autophagy flux in the brain^21,22^.

The first mouse model^21^ is a novel concept that relies on injecting neonatal mice with an adeno-associated virus carrying an mCherry-GFP-LC3 probe. The authors showed the feasibility of using this method for monitoring autophagy flux in neurons of the central and peripheral nervous system using pharmacological stimulation of autophagy in mice. The second model^22^ is the first transgenic mouse recently developed for monitoring autophagy in the CNS. It is based on a ratiometric probe delivered by a Thy promoter and shows abundant fluorescent puncta in different CNS neurons.

Tanida *et al*. published a new method for monitoring autophagy flux that uses a new double fluorescent construct attached to human (h)LC3B^23^. Both fluorescent proteins were selected after a careful screening to ensure they could be used to monitor autophagosomes and autolysosomes. The double fluorescent construct consists of a pH sensitive GFP variant called pHluorin, and a far-red variant of RFP called mKate2 that acts as background fluorescence. Together, pHluorin-mKate2-hLC3B can be used to monitor autophagy flux in cells. In essence, during autophagosome formation, both pHluorin and mKate2 emit fluorescence displaying a merged yellow color. However, during protein degradation in autolysosomes, the internal pH is decreased due to the acidic milieu, and the green fluorescence emitted by pHluorin is rapidly diminished and the fluorescence from mKate2 remains red. Based on this principle, we constructed a new transgenic mouse model called pHluorin-mKate2-hLC3B (PK-LC3).

In the present study, we used PK-LC3 mice to monitor the formation of autophagosomes and autolysosomes in CNS neurons, particularly in Purkinje cells. Using correlative light and electron microscopy (CLEM), we confirmed that fluorescent puncta in cells correspond to autophagosomes and autolysosomes. We utilized stereological analyses to show that autophagosomes and autolysosomes are significantly increased in the perikaryal region of Purkinje cells in PK-LC3 mice following nutrient starvation for 24 hours. Together, our results indicate that autophagy is induced following a brief starvation period. Moreover, it is likely that PK-LC3 mice show great potential as a mouse model for monitoring autophagy in CNS neurons.

## Results

### Generation of PK-LC3 mice and phenotype characterization

**Figure 1:**
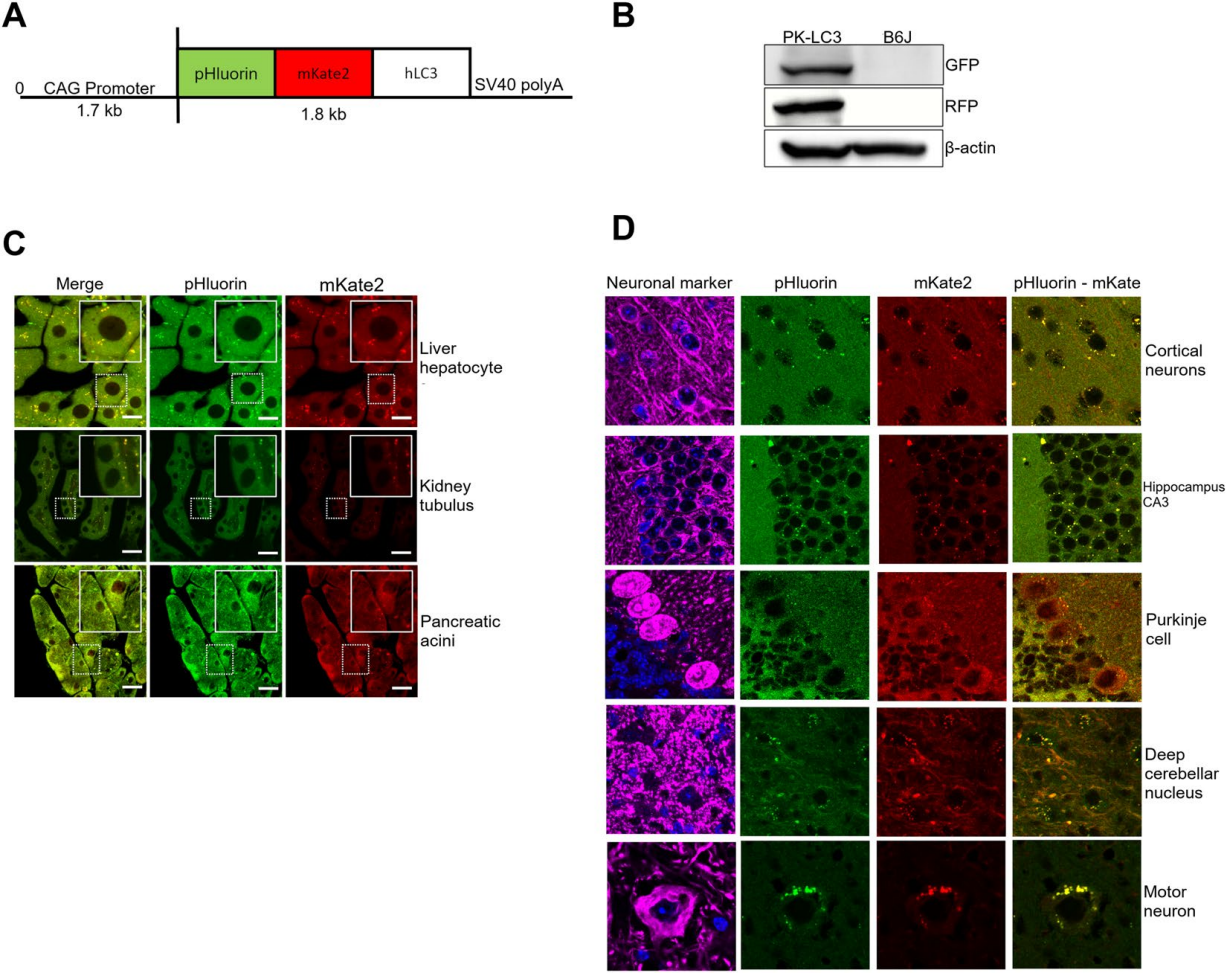
Generation of PK-LC3 mice and phenotype. (**A**) Map for PK-LC3 construct. The CAG promoter is followed by the pHluorin, mKate2 and human LC3B genes. (**B**) Immunoblots from cerebellar lysates of transgenic PK-LC3 and non-transgenic B6J mice. GFP was used to detect pHluorin, RFP was used to detect mKate2 and β-actin as a loading control. (**C**) Images of liver hepatocytes, renal tubular epithelial cells and pancreatic acinar cells from PK-LC3 mice under normal conditions. The left panels show merged images, the central panels show pHluorin, and the right panels show mKate2 signaling. Each area in the dotted boxes is magnified and is displayed within fully lined squares. Scale bar corresponds to 20 μm. D. Stained images of neurons of PK-LC3 mice in normal feeding conditions. Cerebral cortical neurons, hippocampal CA3 neurons, and anterior spinal motor neurons were stained with MAP2 antibody (magenta). Purkinje cells and neurons in the deep cerebellar nucleus were stained with calbindin antibody (magenta). Left panels show merged images, central panels show pHluorin signaling, and right panels show mKate2 signaling. Scale bar corresponds to 10 μm.

Our goal was to develop a transgenic mouse model that could enable *in vivo* monitoring of autophagy and autolysosome activity in CNS neurons. A tandem fluorescent protein fused with human (h) LC3B, pHluorin-mKate2-hLC3B (PK-LC3) was employed to monitor autophagy and autolysosomes in mice. Using the expression plasmid for PK-LC3 under the control of the CAG promoter (Fig. 1A), we generated several mouse colonies and selected one. The mice were fertile, and had no apparent growth defects.

The expression of PK-LC3 was analyzed in the cerebellum of the PK-LC3 mice. Immunoblotting analyses using GFP and RFP antibodies showed that a band at about 70 kDa equivalent to the calculated molecular weight of PK-LC3 (70.5 kDa), was recognized well (Fig. 1B). The characteristic features of fluorescence were then examined in the peripheral tissues and CNS neurons of PK-LC3 mice. In hepatocytes, renal tubular epithelial cells, and pancreatic acinar cells many dot-like structures emitted yellow to orange/red fluorescence, although yellow fluorescent puncta were more prominent in these cells than orange/red ones (Fig. 1C). In CNS tissue, PK-LC3 fluorescent puncta were detected in the perikarya of neurons located in the cerebral cortex, hippocampus, Purkinje layer, and deep nuclei of the cerebellum, as well as in the anterior spinal regions (Fig. 1D). The color of the fluorescent puncta in these neurons varied between yellow and orange/red colors.

### Fluorescent puncta in the CNS increase in response to starvation stress

**Figure 2:**
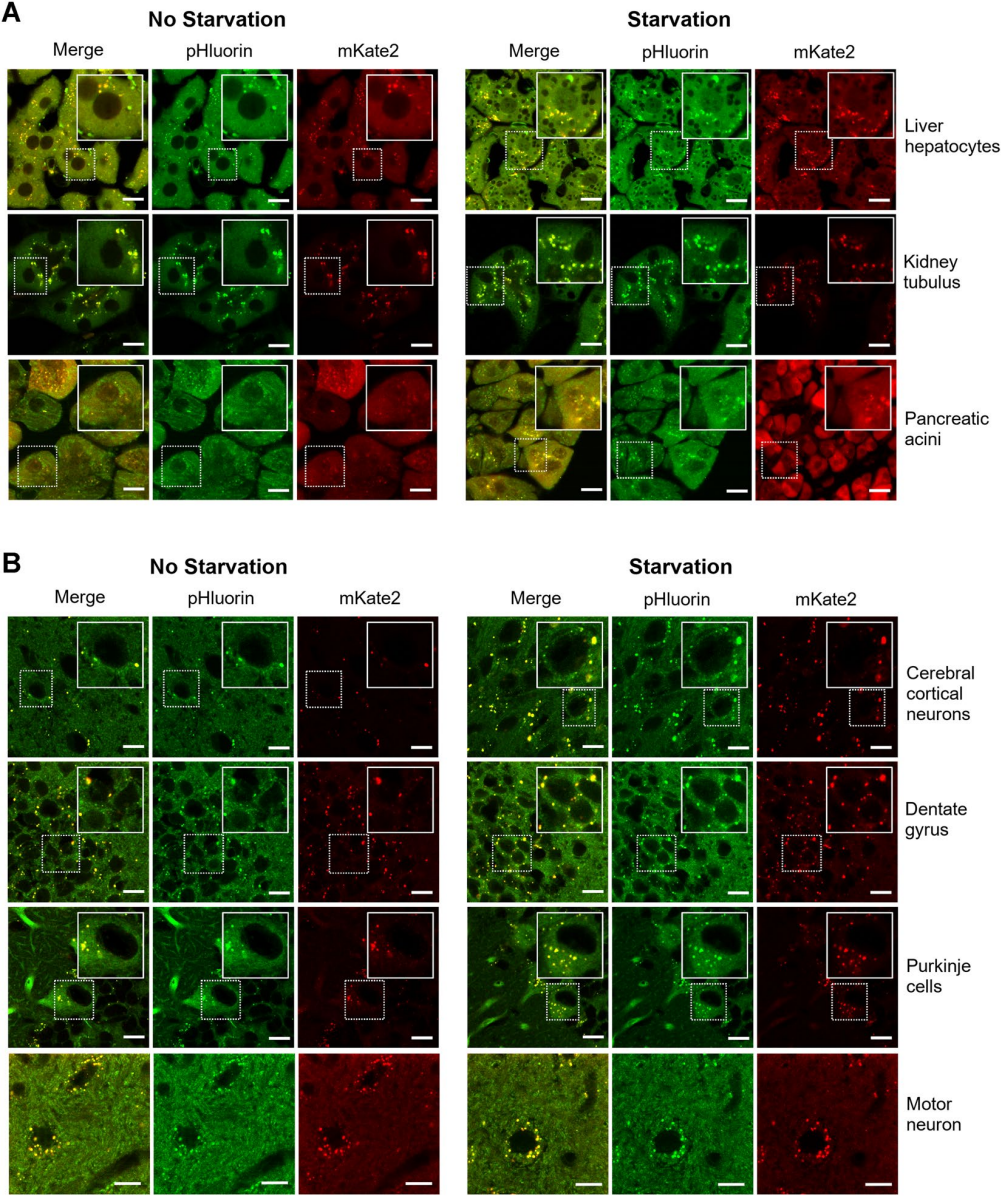
Phenotypes following starvation for 24 hours. (**A**) Images of liver hepatocytes, renal tubular epithelial cells, and pancreatic acinar cells from PK-LC3 mice before and after starvation for 24 hours. Left side panels show tissue images from non-starved mice and right panels show tissue images from mice starved for 24 hours. Each area in the dotted boxes is magnified and displayed within fully lined squares. Scale bar corresponds to 20 μm. (**B**) Images of cerebral cortical neurons, granule cells in the dentate gyrus, cerebellar Purkinje cells, and anterior spinal motor neurons. Left side panels show neuronal images from non-starved mice and right panels show those from mice starved for 24 hours. Each area in the dotted boxes is magnified and displayed within fully lined squares. Scale bar corresponds to 10 μm.

Using PK-LC3 mice, we confirmed that autophagic activity was enhanced in hepatocytes, renal tubular epithelial cells, and pancreatic acinar cells of the mice following starvation for 24 hours (Fig. 2A). In these cells, fluorescent puncta with yellow and orange/red colors became abundant in number. More importantly, PK-LC3 puncta appeared to increase in number in CNS neurons in areas such as the cerebral cortex, hippocampus, dentate gyrus, cerebellum, and spinal cord of the mice following starvation for 24 hours (Fig. 2B). In particular, it was also evident that orange/red puncta were increased in the perikaryal regions of neurons following starvation for 24 hours.

### Characterization of PK-LC3 expression in Purkinje cells

Thus far, we have described how several cell types in PK-LC3 mice exhibited fluorescent puncta that apparently increased in number following starvation for 24 hours. To verify that our mice could be used for monitoring autophagosomes and autolysosomes, we thoroughly analyzed Purkinje neurons before and after starvation for 24 hours using morphometric techniques.

**Figure 3:**
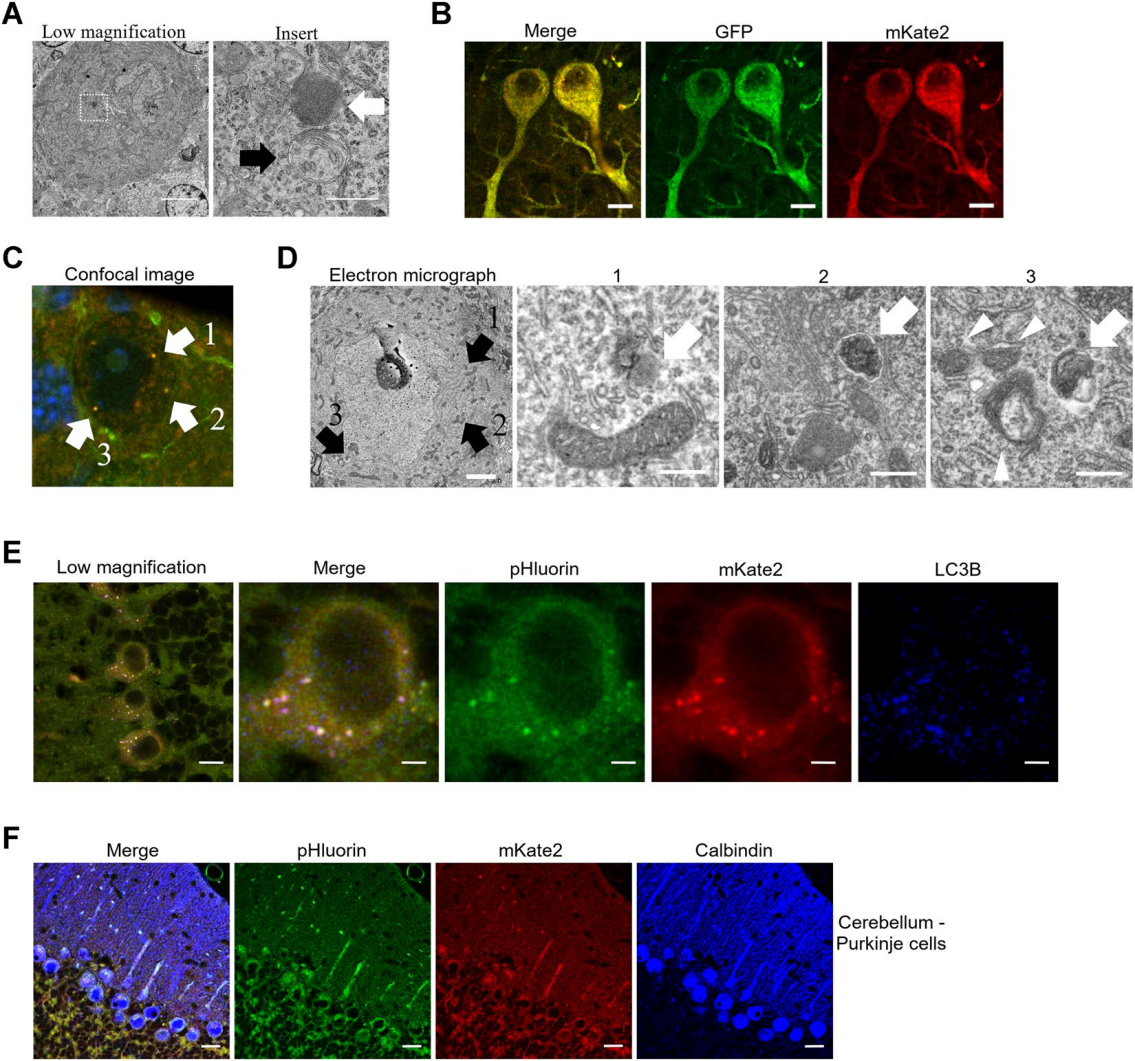
Autophagy and PK-LC3 expression in Purkinje cells. (**A**) Electron micrographs of a Purkinje cell from a B6J mouse. Dotted square in left panel is enlarged and shown in right panel. White arrow indicates a lysosome/autolysosome-like structure and black arrow indicates an autophagosome-like structure. Scale bar corresponds to 2 μm for left panel and 500 nm for right panel. (**B**) Representative images of GFP and RFP immunostaining showing the expression of pHluorin and mKate2 in Purkinje cells of a PK-LC3 mouse. Scale bar corresponds to 10 μm. (**C**) CLEM images of a Purkinje cell from a PK-LC3 mouse. Positive staining for PK-LC3 is marked as 1–3 by white arrows as detected by a confocal laser scanning microscope (left-most panel). Scale bar corresponds to 5 μm. (**D**) Electron microscope image corresponding to areas marked as 1–3 by black arrows (left-center panel). Corresponding areas are shown as high magnification images in images 3D-1 to 3D-3. White arrows indicate 3 different forms of autolysosomes and arrowheads indicate mitochondria. Scale bar corresponds to 5 μm for left panel and 500 nm for panels 1 to 3. (**E**) Immunostaining for LC3B in Purkinje cells from a PK-LC3 mouse. The merged low magnification image shows PK-LC3 puncta that are immunostained for LC3B, exhibiting triple fluorescent puncta for pHluorin and mKate2 and cyan (blue) for LC3B (right-most panel). This confocal image is enlarged in the second right-most panel. The third and fourth panels show pHluorin- and mKate2-positive puncta. The left-most panel shows immunopositive puncta for LC3B (blue color) in Purkinje cells from a PK-LC3 mouse, which are highlighted in white in the merged figures of the right-most two panels. Scale bar corresponds to 10 μm for the low magnification panel and 2 μm for the high magnification panels. (**F**) Representative images of Purkinje cells from a PK-LC3 mouse immunostained with calbindin (blue) antibody. The merged picture and individual pHluorin, mKate2 and calbindin panels are shown from left to right, respectively. Scale bar corresponds to 20 μm.

Purkinje cells from normal B6J mice were first observed by electron microscopy to detect autolysosome/lysosome-like structures or autophagosome-like structures that were encircled by double membranes and contained portions of cytoplasmic organelles (Fig. 3A). Morphological and morphometric analyses revealed no significant difference between the volume densities of autophagosomes and autolysosomes/lysosomes in Purkinje cells of B6J mice when compared to PK-LC3 mice in basal (non-starvation) conditions (Supplementary Fig. A). Using PK-LC3 mice, cerebellar tissue was immunostained with antibodies for GFP and RFP to detect the presence of pHluorin- and mKate2-positive signals. The results clearly demonstrated that both immunosignals of GFP and RFP were clearly expressed in the perikarya and dendrites of Purkinje cells (Fig. 3B).

To further confirm that fluorescent puncta inside Purkinje cell bodies correspond to PK-LC3 puncta, the neurons were analyzed by correlative light and electron microscopy (CLEM). Because GFP is quenched very quickly after the fusion of autophagosomes with lysosomes, we relied on fluorescence by mKate2, a far red RFP variant, which is more resistant to the acidic milieu (degradation in lysosomes). As shown in Fig. 3C, fluorescent puncta in the perikarya of Purkinje cells (Fig. 3C) were confirmed in a corresponding electron micrograph (Fig. 3D) that showed certain areas in the perikaryal region of the neuronal body (Fig. 3D). When these portions were enlarged by electron microscopy, the indicated portions (arrows 1–3) were confirmed to demonstrate three different forms of autolysosomes.

Moreover, we also confirmed that yellow PK-LC3 puncta largely corresponded to positive puncta immunostained for LC3B (Fig. 3E). Since PK-LC3 expressed human LC3, there should be two types of immunostained puncta for LC3: PK-LC3 puncta that were co-stained for LC3B and mouse native LC3 puncta (Fig. 3E). In addition, we used cathepsin D staining to confirm that red puncta correspond to autolysosomes (Supplementary Fig. C). Finally, since the neurons that contained abundant PK-LC3-positive puncta mainly in their perikarya were immunostained for calbindin, they were recognized as Purkinje cells (Fig. 3F).

Together, these results indicated that PK-LC3 puncta correspond to both autophagosomes and autolysosomes in Purkinje cells.

### Characterization of changes in autophagosomes and autolysosomes in Purkinje cells of PK-LC3 mice

**Figure 4:**
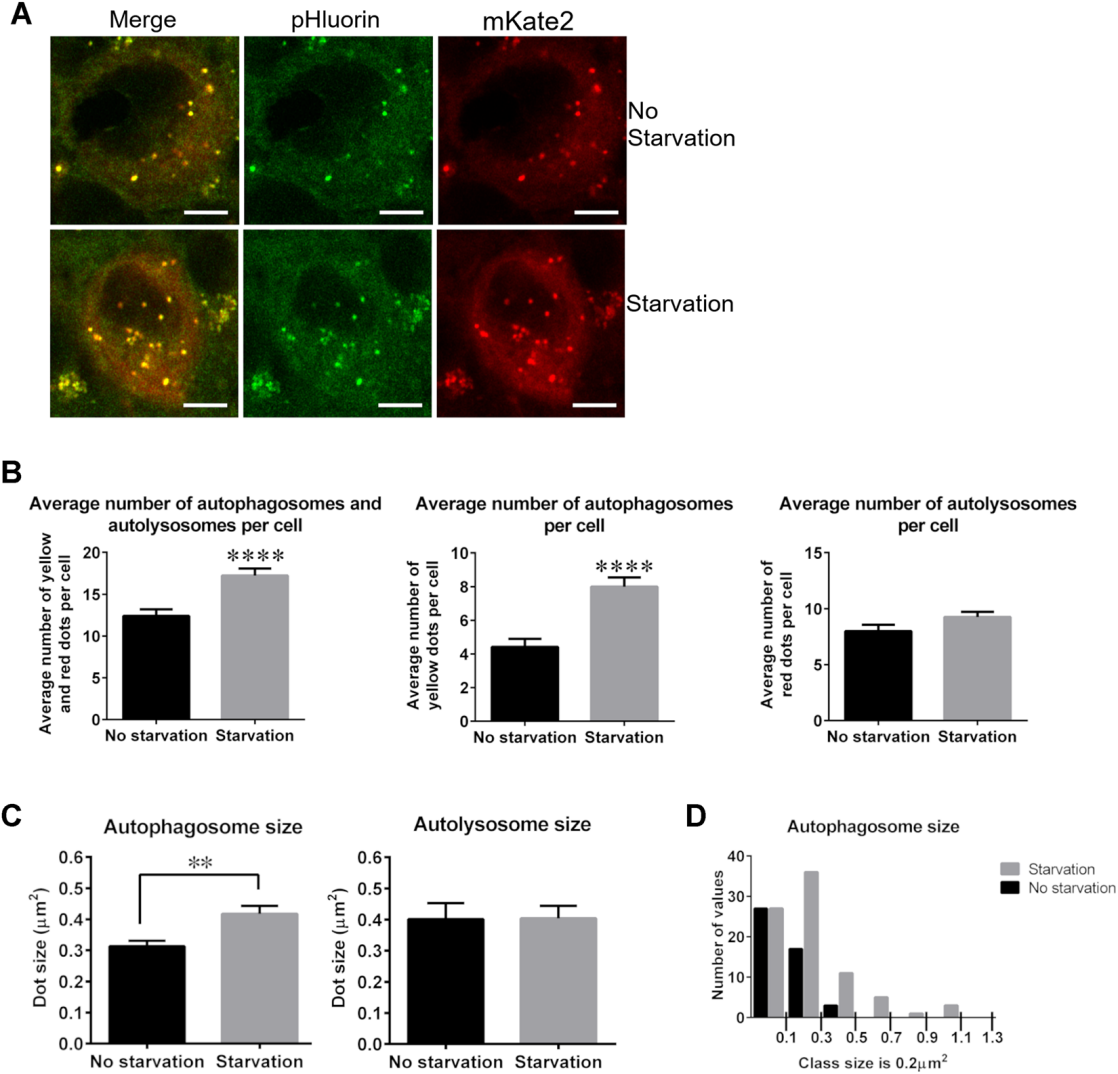
Characterization of autophagy activity in Purkinje cells. (**A**) Representative images of Purkinje cells from PK-LC3 mice before (upper panel) and after (lower panel) starvation for 24 hours. Left panel shows the merged image, central panel shows the pHluorin image and right panel shows the mKate2 image. Scale bar corresponds to 10 μm. (**B**) Quantification of A. Left panel shows bar graphs for the average number of total PK-LC3-positive puncta. Center panel shows average number of yellow PK-LC3 puncta corresponding to autophagosomes, and right panel shows average number of orange/red PK-LC3 puncta corresponding to autolysosomes before and after 24 hours of starvation. The results are expressed as the mean ± SEM. * P < 0.001. (n = 4). (**C**) Bar graphs for average intersected areal size of PK-LC3 puncta in autophagosomes and autolysosomes before and after starvation for 24 hours. **P < 0.001. (n = 3). (**D**) Histogram of intersected areal size of PK-LC3 yellow puncta (autophagosomes) before and after starvation for 24 hours. The number of values occurring in the areal size class from starved mice peaked at a larger class of 0.2–0.4 µm^2^ than those from before starvation (0–0.2 µm^2^). Each class size is 0.2 µm^2^.

To confirm whether the changes in the number of autophagosomes and autolysosomes in Purkinje cells of PK-LC3 mice fluctuate in a nutrient-dependent manner, numbers and densities of fluorescent puncta were analyzed in Purkinje cells of PK-LC3 mice before and after nutrient starvation for 24 hours (Fig. 4A). As for the number of PK-LC3 puncta per intersected perikaryal area, the total number of PK-LC3 puncta (yellow and orange/red) was significantly increased (P = 5.06E-5) following 24 hours of starvation; on average, it was increased 20% following 24 hours of starvation, while the average total number was 12.42 ± 0.7898 (mean ± S.E.) under non-starved conditions and 17.27 ± 0.8174 following starvation for 24 hours (Fig. 4B). Moreover, yellow puncta considered autophagosomes were significantly increased (P = 6.75E-6) following 24 hours of nutrient starvation. Yellow only puncta accounted for less than half of all the total PK-LC3 puncta in both mouse groups: the average number of yellow puncta before starvation was 4.422 ± 0.4825, while it was 8.000 ± 0.5551 following starvation (Fig. 4B). Although an increase in PK-LC3 dot number was detected following nutrient starvation, the changes in the orange/red puncta, considered early autolysosomes, showed no statistical significance (P = 0.0894). The average number before starvation was 8.000 ± 0.5738, while it was 9.267 ± 0.4632 after starvation (Fig. 4B). These results demonstrated that starvation stress for 24 hours increased autophagosome formation in Purkinje cells of PK-LC3 mice.

The average intersected areal size of PK-LC3 puncta corresponding to autophagosomes and autolysosomes in Purkinje cells of PK-LC3 mice was also determined under normal and starvation conditions. The results indicated that the average areal size of autophagosomes was significantly larger (P = 0.0025) in cells following starvation for 24 hours than under normal conditions, whereas that of autolysosomes did not change following starvation (Fig. 4C). The average areal sizes of the puncta for autophagosomes were 0.3034 ± 0.0173 (µm^2^, mean ± SEM) under normal conditions and 0.4039 ± 0.0221 following 24 hours of starvation, whereas those for autolysosomes were 0.3927 ± 0.0457 before starvation and 0.3862 ± 0.0376 after starvation (P = 0.9133). Moreover, the areal size distribution of PK-LC3 puncta in autophagosomes was analyzed under normal and starvation conditions. In the histogram, the number in the areal size class of autophagosomes obtained from PK-LC3 mice before starvation peaked at the class of 0–0.2 µm^2^, it decreased rapidly and appeared in the first three classes (Fig. 4D). On the contrary, the number in the areal size class for the mice following starvation for 24 hours peaked at the class of 0.2–0.4 µm^2^ and continued until the areal size class of 1.0–1.2 µm^2^ (Fig. 4D). These results suggested that nutrient starvation increased not only the number of autophagosomes but also their areal size.

### Electron microscopic morphometry reveals increases in the number of autophagy-related structures in Purkinje cells following nutrient starvation

**Figure 5:**
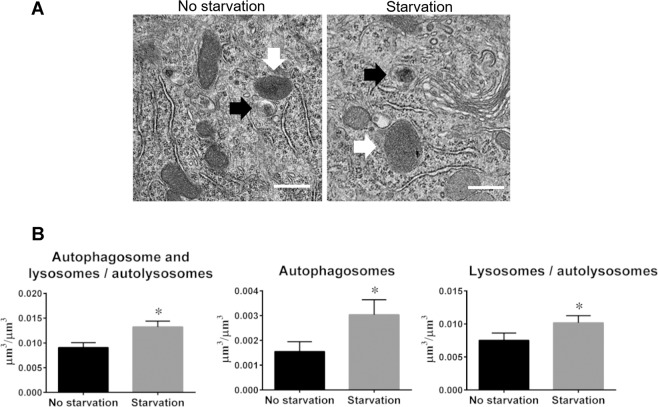
Autophagy activity is increased following starvation for 24 hours. (**A**) Representative electron micrographs of Purkinje cells from PK-LC3 mice. Left image shows a part of the perikaryal region of Purkinje cells of the mice before starvation, while right image exhibits that after starvation. White arrows show lysosomes or autolysosomes, while black arrows display autophagosomes with double membranes. Scale bar corresponds to 500 nm. (**B**) Graphs of the volume densities for autophagy-related structures including autophagosomes and autolysosomes/lysosomes (right panel), autophagosomes (central panel), and autolysosomes/lysosomes (left panel). Left side graph corresponds to all autophagy-related structures. Center graph corresponds to autolysosomes only. Right side graph corresponds to lysosomes and autolysosome structures. The results are expressed as the mean ± SEM. *P < 0.05 (n = 3).

Our previous results indicated that the number of autophagosomes and autolysosomes was increased in Purkinje cells of PK-LC3 mice following 24 hours of starvation. To further understand these results, morphometric approaches at the electron microscopic level were applied to the analysis of these autophagy-related structures in Purkinje cells of the mice before and after starvation. Since it is difficult to distinguish autolysosomes from lysosomes using electron microscopy, the present analysis did not make distinction between the two structures (Fig. 5A). Morphometric results showed that the volume density of all autophagy-related structures (autophagosomes together with lysosomes/autolysosomes) was significantly increased (P = 0.0107) following starvation for 24 hours (Fig. 5B). The mean values for these autophagy-related structures were 0.0091 ± 0.0005 (µm^3^/µm^3^, mean ± SEM) before starvation, and 0.0132 ± 0.0006 after starvation. The volume densities for individual autophagosomes and autolysosomes/lysosomes were also determined in Purkinje cells before and after starvation, and both densities were significantly increased (autophagosomes: P = 0.0309; autolysosomes P = 0.0418) following starvation (Fig. 5B). The mean density for autophagosomes was 0.0075 ± 0.0006 before starvation and 0.0102 ± 0.0006 after starvation, while the average density for lysosomes/autolysosomes was 0.0016 ± 0.0002 before starvation and 0.0030 ± 0.0003 after starvation. It is noted that the increase in the volume densities of lysosomes is was not as pronounced as in those of autophagosomes. Due to a significant increase in the volume density of autophagosomes in Purkinje cells following starvation for 24 hours, our results strongly support that autophagy is induced in CNS neurons in a nutrient starvation-dependent manner.

Using both light and electron microscopy, our results provide evidence that the number of autophagosomes and autolysosomes is increased in Purkinje cells in a nutrient-dependent manner.

## Discussion

The importance of autophagy has been demonstrated in CNS tissues. Studies using mouse models of core autophagy proteins^24^ atg5^5^, atg7^6^, and atg9^7^ deficiency in neurons have all shown similar aggressive pathological phenotypes that result in early death. Other mouse models that target lysosomal degradation in CNS neurons, thereby affecting autophagy-lysosomal degradation, also exhibit neuropathological phenotypes^25–27^. In humans, there is evidence that many important neurodegenerative diseases are somehow connected to autophagy and lysosomal dysregulation^11,14,28,29^. Considering all data currently available, it can be demonstrated that there is a strong link between a dysregulated autophagy-lysosomal degradation system and neuropathology.

It has been shown that autophagy flux is tissue-dependent when starved GFP-LC3 mice are used. In CNS neurons, autophagosome number has also been demonstrated to increase in a nutrient-dependent manner by using the mice^18^. Although GFP-LC3 puncta largely correspond to autophagosomes and the early phase of autolysosomes, fluorescence due to GFP disappears soon after fusion with lysosomes^30^. Using neonatal mice injected with mCherry-GFP-LC3 carrying an adeno-associated virus, yellow and red fluorescence increases in anterior spinal motor neurons after stimulation by rapamycin or trehalose, but the yellow portion (autophagosomes) is reduced and shifts to red (autolysosomes) following stimulation. These results indicate that autophagy in CNS neurons occurs in a manner similar to that seen in somatic tissue cells, while its flux is increased after stimuli not only by starvation but also by rapamycin/trehalose.

During the preparation of the present manuscript, a paper was published describing a genetic mouse model for monitoring autophagy. These new TRGL6^22^ mice were developed by Lee *et al*. One significant difference between PK-LC3 mice and TRG6L mice is in the design of the model. The promoter used by TRGL6 transgenic mice is Thy1, which is different from the CAG promoter used in PK-LC3 mice. How much this affects the expression of the fluorescent probe in both models is unknown at this point. A common phenotype trait in both PK-LC3 mice and TRGL6 mice appears to be the robust expression of fluorescent puncta in several areas of the CNS. A full comparison between both models is complicated because of the very different methods used for validating each model. For our study, we decided to validate the feasibility of PK-LC3 mice for monitoring autophagy flux using a combination of fluorescence microscopy and electron microscopy. In the study to validate TRGL6 mice, the researchers relied on a combination of *in vivo* and *in vitro* studies along with pharmacological modulation for evaluating autophagy flux. At this time, the full extent of the differences between TRGL6 and PK-LC3 mice is unknown. More studies need to be done with both mice to understand each of the mouse models advantages and shortcomings.

Focusing on the results from the studies using TRGL6, PK-LC3, GFP-LC3 transgenic mice, along with the AAV injection model of mCherry-GFP-LC3, we can conclude that it is a positive development that more tools are available for investigating neuronal autophagy. Some common observations made in all of the aforementioned models have revealed important features regarding autophagy in the CNS. For example, all mouse models have shown evidence that neurons in different regions of the brain show detectable levels of basal autophagy activity. These models have also shown that autophagy activity in the CNS is sensitive to stimulation. The ability to observe such changes *in vivo* will be very important for studies focusing on understanding how neuropathological phenotypes develop as a result of dysregulated autophagy in the CNS.

To develop a mouse model for monitoring autophagy in CNS neurons, we established a transgenic mouse line expressing PK-LC3, based on a concept established in a study by Tanida *et al*.^23^. As stated in the Results section, CLEM evidence showed we demonstrated that PK-LC3 puncta corresponded to autophagosomes (yellow puncta) and autolysosomes (orange/red puncta). Following a period of 24 hour starvation, only yellow puncta increased significantly in number in a nutrient starvation-dependent manner, in contrast orange/red puncta, indicating autolysosomes, exhibited an increasing tendency but did not significantly change after following starvation. Since pHluorin in PK-LC3 is much more sensitive to an acidic milieu than GFP, it is possible to assume that the time period of each yellow dot as an autophagosome in PK-LC3 neurons was much shorter than that in either GFP-LC3 or mCherry-GFP-LC3 neurons. That result indicates that the yellow puncta in PK-LC3 neurons were more promptly translated to orange/red puncta than those in the mCherry-GFP-LC3 soon after fusion with lysosomes. Another consideration is that the fluorescence of the yellow portion in the mCherry-GFP-LC3 mouse motor neurons was decreased after stimuli by rapamycin or trehalose. The present data, however, indicated that starvation stress to the PK-LC3 mice for 24 hours increased not only the number of yellow puncta for autophagosomes but also increased the individual size of autophagosomes in Purkinje cells, as evidenced by the histogram of the areal size of yellow puncta (Fig. 4D). These results could further suggest that autophagosome formation is continuously stimulated by nutrient starvation, which may differ from chemical stimulation by either rapamycin or trehalose. When considering the pH sensibility of pHluorin and the absence of a significant increase in autolysosome number in Purkinje cells following starvation, we speculate that translation of autophagosomes to autolysosomes likely proceeds slowly. Taken together, the data of the present study are the first to clearly show the induction of autophagy, in particular autophagosomal formation, in CNS neurons of PK-LC3 mice following starvation for 24 hours.

In addition to the presence of autophagosomes and autolysosomes in cerebellar Purkinje cells, they were also observed in cerebral cortical neurons, hippocampal neurons, granule cells in the dentate gyrus, and in anterior spinal motor neurons. Moreover, our findings that fluorescent puncta in Purkinje cells corresponded to autophagosomes and autolysosomes (early autolysosomes) were confirmed by LC3 immunostaining and the results of CLEM. Fluorescence microscopic analyses showed an increased number of autophagosomes and autolysosomes, although the change in the number of autolysosomes was not statistically significant. Changes in these cytoplasmic organelles in Purkinje cells of PK-LC3 mice by starvation for 24 hours were also confirmed by stereological approaches at the electron microscopic level, particularly concerning those in autophagosomes. It is well known that electron microscopy hardly distinguishes between autolysosomes and lysosomes. Measurement of these structures was performed by treating them as lysosomal structures whose volume density significantly changed following starvation. Morphometric analyses by fluorescent and electron microscopy revealed that the number, areal size and volume density of autophagosomes all were significantly changed in a nutrient starvation-dependent manner.

Finally, the present study using PK-LC3 mice demonstrated that autophagy activity was stimulated in CNS neurons, particularly in Purkinje cells, in a nutrient starvation-dependent manner. In fact, our analyses clearly showed that increased autophagy activity in Purkinje cells following starvation was demonstrated not only by the increase in autophagosome number, but also by the translation of autophagosomes to autolysosomes/lysosomes. This translation appears to proceed slowly and will be investigated in future studies. As far as we could ascertain, PK-LC3 mice represent a viable alternative model for monitoring the autophagy/lysosomal system at least in Purkinje cells, as evidenced by light and electron microscopic results.

## Materials and Methods

### Animals

PK-LC3 mice were generated using a previously published method^31^. Briefly, sperm with pEX-PK-hLC3^23^ were arranged and injected into oocytes obtained from C57BL/6 J mice (Charles River Laboratories Japan). The oocytes were incubated and two-cell stage embryos were transferred to the oviducts of pseudopregnant ICR recipient females (Charles River Laboratories, Japan). The transfer of 219 embryos produced 19 offspring (8.7%). After extraction of DNA from tail biopsies, 19 mice were screened by PCR analysis for incorporation of the transgene, using the forward primer CMV-1(5′-GGCTTCTGGCGTGTGACC-3′) and the reverse primer CMV-2 (5′-AGCCACCACCTTCTGATAG-3′). Two mice positive for the transgene were used as founders. These F0 mice were mated with C57BL/6 J mice and were maintained as heterozygotes for the PK-LC3 transgene. One of the transgenic lines, PK-LC3#301, was used for all the experiments described in this study.

The procedures involving animal care and sample preparation were approved by the Animal Experimental Committee of the Juntendo University Graduate School of Medicine, and were performed in accordance with the NIH guidelines and the regulations and guidelines for the care and use of laboratory animals of the Juntendo University Graduate School of Medicine.

### Antibodies

The following primary antibodies used in the present study were purchased from commercial companies: rabbit anti-green fluorescent protein (MBL - #598), goat anti-red fluorescent protein (MBL - #PM005), goat anti-calbindin (Frontier Science - #AF1840), rabbit anti-calbindi (Frontier Science - # Calbindin-Rb-Se-1), guinea pig anti-MAP2 (Synaptic Systems #188-004), rabbit anti-cathepsin D [EPR3057Y] (Abcam - #75852), and mouse monoclonal anti-LC3B (Cell Signaling - #3868).

### Immunoblotting

Cerebellar tissues were extracted from PK-LC3 and B6J mice at 8 weeks of age and homogenized in N-Per lysis buffer (Thermo - #87792) using a Dounce homogenizer. Lysates were cleared by centrifugation at 10,000 *g* at 4 °C for 10 minutes. The protein concentration was determined using a Pierce BCA protein assay kit (Thermo - #23228). Immunoblots were carried out as described previously^7^. For SDS page, 20 µl of protein was applied and resolved in an acrylamide gel. Afterwards, the membranes were transferred to a Fluorotrans PVDF membrane (Pall Life Sciences - #79548 A). After blocking with 5% nonfat milk for 1 hour, the membranes were incubated with primary antibodies overnight at 4 °C, and then with horse radish peroxidase (HRP)-conjugated secondary antibodies for 1 hour. Immunoreactivity was determined using Super West Dura Extended duration substrate reagent (Thermo - #34076). Each protein band was detected and analyzed using the Fusion FX western blot imaging system (Vilber Lourmat).

### Sampling procedures for morphological analyses

Sample preparation for light and electron microscopy was performed as previously described^7^. Deeply anesthetized mice were fixed by cardiac perfusion with 4% paraformaldehyde in 0.1 M phosphate buffer (PB) (pH 7.4) for light microscopy. Cerebellar tissues were excised from the mice and postfixed with the same fixative for 24 hours. Afterwards, the tissues were cryoprotected using a series of 15, 20 and 30% sucrose solutions. Finally, the tissue samples were embedded in OCT compound (Sakura #4583) and frozen using liquid nitrogen. Frozen sections were cut to a thickness of 8 µm using a cryostat (Leica CM3050S). For electron microscopy, deeply anesthetized mice were perfused transcardially with 2% glutaraldehyde-2% PFA in 0.1 M PB. Samples were postfixed in the same fixative at 4 °C overnight and then fixed with 1% OsO4 in 0.1 M PB buffer. Fixed tissues were further processed and embedded in Epon 812 epoxy resin (Oken shoji). Silver sections were cut with an ultramicrotome (Leica Ultracut UC7, Leica microsystems), stained with uranium acetate and lead citrate, and observed using a Hitachi HT7700 microscope.

### Immunostaining

Frozen sections of PK-LC3B mouse cerebellar tissues were immunostained according to a previously described method^7^. Briefly, after blocking with TNB buffer (Perkin Elmer), sections were incubated with primary antibodies at 4 °C overnight, followed by 1 hour of incubation with fluorescently labeled anti-goat, anti-mouse, or anti-rabbit secondary antibodies (Jackson Immuno Research). For double immunostaining, frozen sections were treated with anti-GFP and anti-RFP overnight at 4 °C and further incubated with fluorescently labeled anti-rabbit and anti-mouse secondary antibodies for 1 hour. Samples were viewed with a confocal LSM880 microscope (Zeiss).

### PK-LC3 dot quantification

To quantify PK-LC3 puncta in Purkinje cells, cerebellar samples from 4 mice under normal fed conditions and from 4 mice starved for 24 hours were analyzed. The total number of fluorescent puncta from a total of 10 Purkinje cells per mouse were counted using Image J software (US National Institute of health). The samples were observed using a confocal LSM880 microscope (Zeiss).

### PK-LC3 dot size analysis

To quantify PK-LC3 dot size in Purkinje cells we used cerebellar samples from a group of 3 mice in normal feeding conditions and from a group of 3 mice starved for 24 hours. For each mouse we analyzed 10 Purkinje cells for a total of 30 Purkinje cells per group. Image J software was used to analyze the total area for yellow puncta corresponding to autophagosomes and orange/red puncta for autolysosomes. The average area per dot for both autophagosomes and autolysosomes under normal and starved conditions was analyzed first. Then, from these data, a histogram was produced for determining the frequency distribution of the total number of values of autophagosomes, according to their size. Each class size corresponds to a 0.2 µm^2^ interval.

### Morphometry

Six PK-LC3 mice were used and divided into two groups: normal feeding and 24-hour starved mouse groups. Ten Purkinje cells per mouse for a total of 30 different Purkinje cells per group were randomly selected. To cover an entire Purkinje cell area, about 7–10 fields at 4000X magnification were analyzed. Using a previously described^32^ point counting method, the perikaryal volume density of autophagosomes and autolysosomes/lysosomes was calculated. The parameters were measured at 3.3 times the original magnification using a double lattice system of 2.5 cm spacing that was superimposed on each of the perikaryal fields of Purkinje cell bodies in a computer display. Using these data, the volume density of all target structures (autophagosomes and autolysosomes/lysosomes) in each Purkinje cell was calculated first. Then, the volume density of autophagosomes and lysosomes/autolysosomes were calculated separately. Since lysosomes and autolysosomes are difficult to distinguish ultrastructurally, these structures were counted and calculated together.

### Correlative light and electron microscopy (CLEM)

The precise localization of PK-LC3B was analyzed following a previously described method^33^. Briefly, after perfusion fixation as described above, the specimens were cut into 30 µm sections using a vibratome (Leica, VT1200S), and were stained with DAPI for 1 hour at room temperature. After washing with PBS, sections were mounted on a grid glass-bottom dish (Matsunami - #D11130H), and then were observed using a confocal laser microscope (Zeiss, LSM880). Sections were postfixed with 2% glutaraldehyde-1% OsO_4_, dehydrated, and embedded in Epon 812 epoxy resin (Oken shoji). Thin sections were cut at 80 nm with an ultramicrotome (Leica Ultracut UC7, Leica microsystems), stained with uranyl acetate and lead citrate, and observed with a scanning electron microscope (SEM) (Helios NanoLab 660, FEI). To determine the perikaryal site of Purkinje cells that corresponded to mKate2 (red) fluorescent sites, positive fluorescent sites in a Purkinje cell were superimposed on the SEM figure of the corresponding cell, and exact organelles were identified in the SEM figure.

### Statistical analysis

Statistical analysis was performed using Graphpad Prism software (MDF). A student’s T test was used to analyze PK-LC3 fluorescent dot quantification, dot size analysis, and morphometry analysis. Results are expressed as the mean ± the standard error of the mean (SEM).

## Supplementary information

Supplemental information.
