# Supplementary material for: Characterization of starvation-induced autophagy in cerebellar Purkinje cells of pHluorin-mKate2-human LC3B transgenic mice
Source: Sci Rep. 2020 Jun 15;10:9643. doi: 10.1038/s41598-020-66370-6 (PMC7295967; doi:10.1038/s41598-020-66370-6)
Supplement: Supplementary file 1 — Supplemental information. [file 41598_2020_66370_MOESM1_ESM.docx]

Supplementary information

Title:

Characterization of starvation-induced autophagy in cerebellar Purkinje cells of pHluorin-mKate2-human LC3B transgenic mice

Authors:

Juan Alejandro Oliva Trejo, Isei Tanida^*^, Chigure Suzuki, Soichiro Kakuta, Norihiro Tada, and Yasuo Uchiyama^*^

*Corresponding authors

**Supplementary figures and legends**

**Supplementary Fig. A.**


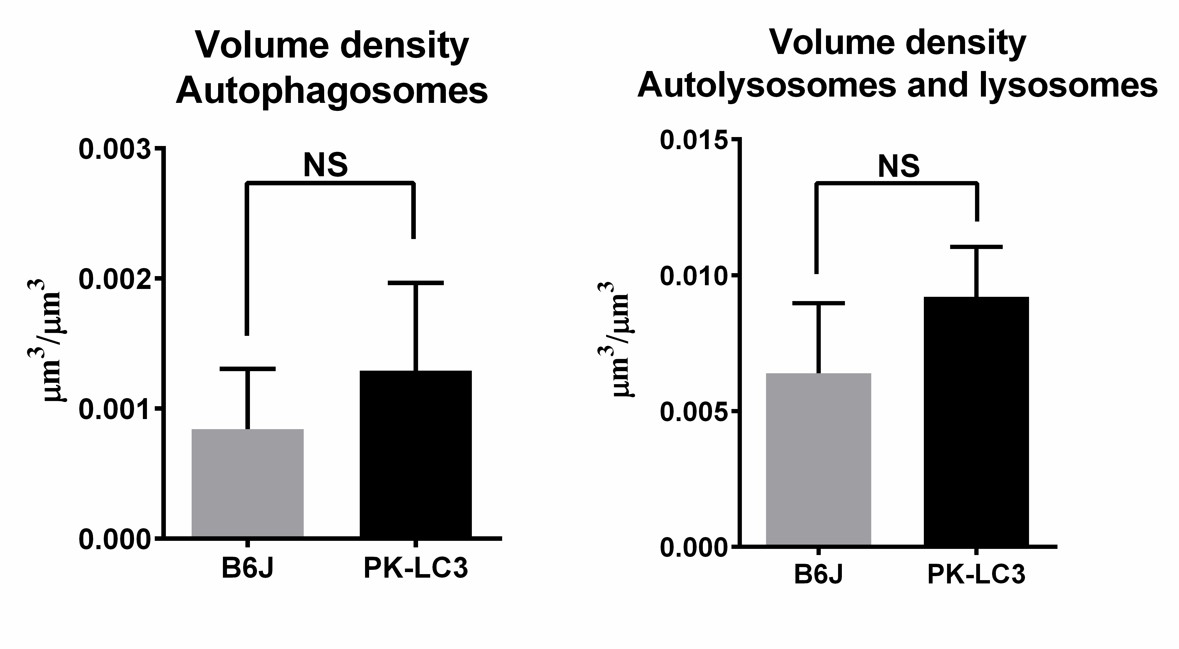


**Supplementary Fig. A**

We compared 3 mice for B6J and 3 mice for PK-LC3 groups. We analyzed 5 electron micrographs per mice for a total of 15 electron micrographs per group. Results analyzed by Student’ T test showed no significant statistical difference between both groups (autophagosomes = P < 0.6119; autolysosomes = P < 0.42). Volume density for B6J mice autophagosomes was 0.0008427±0.0004624 and for PK-LC3 mice was 0.001292±0.0006743 (mean±SEM). Volume density for B6J mice lysosomes/autolysosomes was 0.006391±0.002577 and for PK-LC3 mice was 0.009202±0.001840.

**Supplementary Fig. B.
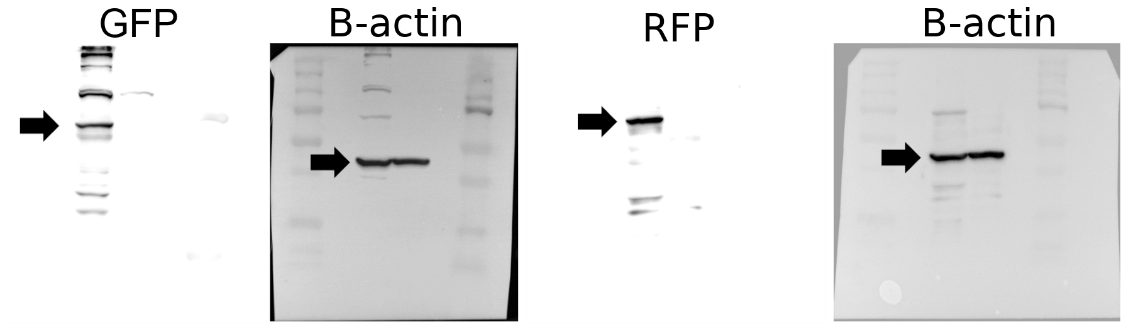
**

**Supplementary Fig. B.**

Whole membrane images from immunoblots used for analyzing GFP, actin for GFP membrane, RFP, and actin for RFP.

**Supplementary Fig. C.**

**
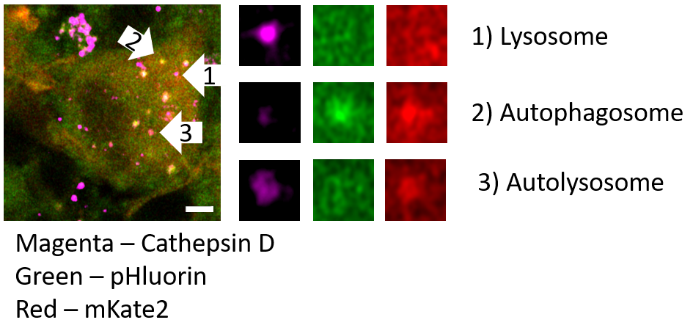
**

**Supplementary Fig. C.**

Representative Purkinje cell from PK-LC3 mice stained with cathepsin D antibody (left side panel). Arrows point to selected structures corresponding to: 1) lysosome, 2) autophagosome, and 3) autolysosome. Selected structures are analyzed using 3 color filters in right side panels. Magenta color corresponds to cathepsin D, green corresponds to pHluorin, and red color corresponds to mKate2. Scale bar corresponds to 2μm.
